# Differentiation and Transmigration of CD4 T Cells in Neuroinflammation and Autoimmunity

**DOI:** 10.3389/fimmu.2017.01695

**Published:** 2017-11-29

**Authors:** Sandip Ashok Sonar, Girdhari Lal

**Affiliations:** ^1^National Centre for Cell Science, Pune, India

**Keywords:** blood–brain barrier, experimental autoimmune encephalomyelitis, CD4 T cells, neuroinflammation, transendothelial migration

## Abstract

CD4^+^ T cells play a central role in orchestrating protective immunity and autoimmunity. The activation and differentiation of myelin-reactive CD4^+^ T cells into effector (Th1 and Th17) and regulatory (Tregs) subsets at the peripheral tissues, and their subsequent transmigration across the blood–brain barrier (BBB) into the central nervous system (CNS) parenchyma are decisive events in the pathogenesis of multiple sclerosis and experimental autoimmune encephalomyelitis. How the Th1, Th17, and regulatory Tregs transmigrate across the BBB into the CNS and cause CNS inflammation is not clearly understood. Studies with transgenic and gene knockout mice have unraveled that Th1, Th17, and Tregs play a critical role in the induction and resolution of neuroinflammation. However, the plasticity of these lineages and functional dichotomy of their cytokine products makes it difficult to understand what role CD4^+^ T cells in the peripheral lymphoid organs, endothelial BBB, and the CNS parenchyma play in the CNS autoimmune response. In this review, we describe some of the recent findings that shed light on the mechanisms behind the differentiation and transmigration of CD4^+^ T cells across the BBB into the CNS parenchyma and also highlight how these two processes are interconnected, which is crucial for the outcome of CNS inflammation and autoimmunity.

## Introduction

Homeostasis of central nervous system (CNS) is maintained by various mechanisms operating in both the CNS and the peripheral immune system. Due to the presence of barriers, CNS antigens are not exposed to cells of the peripheral immune system, which ensures a lack of effector immune response to CNS antigens in the steady state (1). However, upon recognition of CNS-derived antigens or cross-reactive microbial antigens, the peripheral CD4^+^ T cells have escaped from the central tolerance, mount a robust immune response, and infiltrate into the CNS. Such infiltration of CD4^+^ T cells causes CNS autoimmune diseases such as multiple sclerosis (MS) and experimental autoimmune encephalomyelitis (EAE) (2). MS is a human autoimmune demyelinating disease of the CNS characterized by massive infiltration of inflammatory lymphocytes and myeloid cells into the brain and spinal cord, leading to demyelination, axonal damage, and loss of neuromuscular functions (3). Most of the clinical and pathological features of MS are recapitulated in the animal model, EAE, which is one of the important models used to study CNS inflammatory diseases. EAE is also used for evaluating the efficacy of several therapeutic strategies to control neuroinflammation and autoimmunity (3).

## Activation of Myelin-Specific CD4^+^ T Cells During CNS Inflammation and Autoimmunity

There is a long-standing hypothesis in the field that the activation of myelin-specific CD4^+^ T cells requires a trigger from some environmental factors (4). EAE is induced by activating myelin-reactive lymphocytes through peripheral immunization with myelin antigens. EAE can also be induced in susceptible animal hosts either by subcutaneous immunization (s.c.) with myelin antigens emulsified in complete Freund’s adjuvant (CFA) or by the adoptive transfer of *in vitro*-activated myelin-specific CD4^+^ T cell subsets such as Th1 and Th17 (5, 6). Among the various myelin proteins, proteolipid protein, myelin basic protein, and myelin oligodendrocyte glycoprotein (MOG), and their corresponding immunodominant peptides have been extensively used to induce EAE in different rodent hosts (2, 5). However, this also requires administration of pertussis toxin, highlighting the importance of environmental factors in the development of CNS pathology (7, 8). CD4^+^ and CD8^+^ T cells that have low affinity/avidity for myelin antigens, escape thymic selection, and are localized mainly to the secondary lymphoid organs, where they remain in the tolerant state under homeostatic conditions (9). The subcutaneous deposition of myelin peptide emulsion attracts and activates professional antigen-presenting cells (APCs), such as dendritic cells, macrophages, and B cells, at the site of injection. These APCs take up the antigens and migrate to the draining lymph nodes, where they process and present antigenic peptides to the T lymphocytes. Immunization (s.c.) of C57BL/6 mice with MOG_35–55_-CFA emulsion along with intravenous pertussis toxin were found to induce antigen-specific Th1 and Th17 cells in the draining lymph nodes, and at the same time limit the regulatory T (Treg) number and function (7, 10). Interestingly, T-cell receptor (TCR)-transgenic mice, such as 2D2 mice in which CD4^+^ T cells are engineered to express MOG_35–55_-specific TCR, develop spontaneous CNS autoimmunity (11), suggesting the importance of CD4^+^ T cells in EAE. By using several knockout and transgenic mice, molecules involved in the TCR and costimulatory and coinhibitory signaling in the activation, proliferation, and differentiation of myelin-specific CD4^+^ T cells have been evaluated. Furthermore, several members of the TNF-receptor superfamily critically regulate the CD4^+^ T cell response both in the secondary lymphoid organs and inflamed CNS and perturb the pathology of EAE (12).

## Differentiation of Myelin-Specific CD4^+^ T Cells

**Figure 1:**
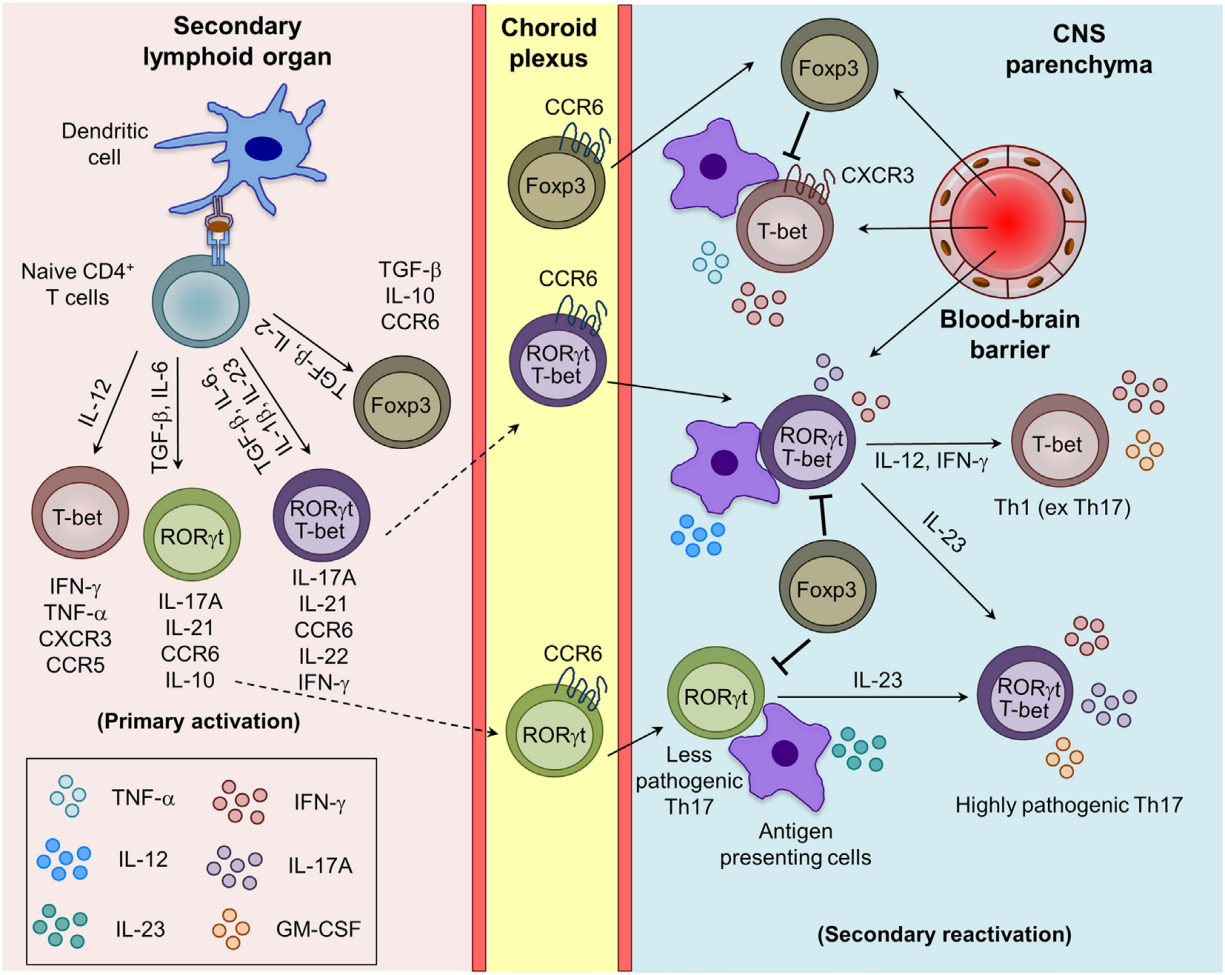
Generation of myelin-reactive effector Th1, Th17, and regulatory iTreg cells and their plasticity in the central nervous system (CNS) parenchyma during experimental autoimmune encephalomyelitis. Upon appropriate myelin antigen presentation, and in the presence of adequate costimulatory molecules and cytokine signaling, naive CD4^+^ T cells are activated and give rise to effector (Th1 and Th17) and regulatory (iTreg) T cells. Th17 cells are trafficked into the CNS mainly through the choroid plexus using CCR6–CCL20 interactions, whereas Th1 cells cross the blood–brain barrier mostly using CXCR3–CXCL9/10/11 interactions. The reactivation of the infiltrating Th1 and Th17 cells by the local antigen-presenting cells (APCs) in the CNS boosts the cytokine secretion and pathogenic potential of Th1 and Th17 cells. Under the influence of IL-12 and IL-23 produced by the APCs, Th17 cells acquire the Th1 (ex Th17) and highly pathogenic Th17 (RORγt^+^T-bet^+^) phenotype.

The naive CD4^+^ T cells, when stimulated by myelin APCs and specific cytokines, differentiate into various effector and regulatory lineages (Figure 1). Th1 cells secrete IFN-γ and TNF-α and are critical for controlling intracellular pathogens and induction of delayed-type hypersensitivity response. Excess activation of Th1 is involved in many organ-specific inflammations, including MS and EAE (13). In the presence of a strong TCR signal, IL-12/STAT4 and IFN-γ/STAT1 signaling induces the Th1-specific transcription factor T-bet, which amplifies IFN-γ/STAT1/T-bet signaling and drives Th1 differentiation (14, 15). Furthermore, T-bet cooperatively interacts with other transcription factors such to RUNX1, RUNX3, GATA3, IRF4, and BCL6 to inhibit the differentiation of alternative CD4^+^ T cell subsets (16, 17). Mice deficient in Th1-associated factors such as T-bet and STAT4 are resistant to the development of EAE (18), whereas IFN-γ^−/−^, IFN-γR^−/−^, and STAT1^−/−^ mice develop more severe EAE (19). This suggests that Th1 cells play a critical role in the pathogenesis of EAE and MS through diverse mechanisms.

In humans and mice, various cytokines induces the differentiation of Th17 cells with diverse phenotypes and functions. The conventional Th17 cells generated in the presence of TGF-β1 and IL-6 are non-pathogenic during EAE, being involved in the maintenance of mucosal surface homeostasis and anti-bacterial defense (20, 21). However, several other factors such as IL-1β, IL-23, and TGF-β3 have been identified to favor the generation and maintenance of highly pathogenic Th17 cells during EAE (21–24). Mice deficient in IL-23 or IL-23R are completely resistant to the development of EAE (23, 25). Th17 cells that coexpress RORγt and T-bet and produce both IL-17A and IFN-γ, are highly pathogenic and preferentially recruited into the CNS, suggesting that T-bet enhances the pathogenicity of Th17 cells (13, 21, 26). The detailed development, phenotypic, and functional differences between pathogenic and non-pathogenic Th17 cells have been reviewed recently (21). Interestingly, a recent report shows that IL-23 induces a switch from CCR6 to CCR2 usage and controls the development and migration of highly encephalitogenic granulocyte macrophage-colony stimulating factor (GM-CSF)-expressing Th17 cells into the CNS, suggesting that homing receptors and pathogenic functions are imprinted during differentiation (27). It has been shown that IL-12 induces STAT4 signaling and also triggers GM-CSF expression in Th1 cells and promote EAE development (28). Interestingly, GM-CSF-producing Th1-like cells are also found in the cerebrospinal fluid (CSF) of MS patients (29), suggesting that GM-CSF may contribute to the pathogenic function of Th17, IFN-γ expressing ex-Th17 and Th1 cells. The chronic inflammatory signals can affect the transcriptional and/or epigenetic signature and control the plasticity of Th1, Th17, and Tregs in the inflamed CNS and lymphoid organs during EAE (30). The IL-23-induced alternatively activated Th17 (T-bet^+^RORγt^+^) or ex-Th17 cells that acquire T-bet and IFN-γ and express negligible RORγt and IL-17 are more pathogenic (31, 32). Similarly, transdifferentiation of Th17 into Tregs, and Tregs to effector Th1, Th2, and Th17 cells are also known (33, 34). IL-23-induced phosphorylation and nuclear localization of STAT3/STAT4 heterodimer has been shown to control the generation of encephalitogenic Th1/Th17 cells (35). More studies are needed to understand the minimum essential cytokine stimuli is require to generate highly pathogenic Th17 cells and that may help in designing better therapeutic strategies to control the inflammation and autoimmunity.

Regulatory T cells are suppressive in nature and known to control the myelin-reactive CD4^+^ effector T cell response and are therefore pivotal in regulating CNS inflammation during EAE (36, 37). Based on their developmental pathways, they are classified as natural Tregs (nTrgs; thymic-derived) or induced Tregs (iTregs; extrathymic-derived) (34, 38, 39). nTregs express Foxp3, a lineage-defining transcription factor, and CD25 (IL-2Rα) during their development in the thymus. iTregs are generated from naive CD4^+^ T cells in the presence of TGF-β in the peripheral lymphoid tissue, which induces the expression of Foxp3 through STAT5 activation (40, 41). In both humans and mice Tregs, Foxp3 required for their suppressive capacity, and its deficiency is associated with the development of spontaneous autoimmunity (38, 39, 42). Tregs employ diverse contact-dependent (expression of CTLA4, FasL, and LAG3) and contact-independent (secretion of TGF-β and IL-10, deprivation of IL-2, and ectonucleotidases CD39/CD73-mediated conversion of extracellular inflammatory ATP/ADP into adenosine) mechanisms to inhibit the functions of myelin-reactive pathogenic T cells and other effector myeloid cells (43). The generation of myelin antigen-reactive CD4^+^ T cell subsets and the plasticity of Th1 and Th17 cells in inflamed CNS during EAE are depicted in Figure 1.

Other subsets of CD4^+^ T cells, such as Th9, T follicular helper (Tfh), T follicular regulatory, and T regulatory 1 (Tr1), are also reported to contribute to the development of neuroinflammation and autoimmunity. The adoptive transfer of MOG-specific Th9 cells is known to induce EAE in C57BL/6 recipients (44). Moreover, IL-9 is required for mast cell activation, which has previously been shown to degrade myelin during CNS inflammation (45). Tfh cells, which are mainly involved in the regulation of germinal center reaction, are also hypothesized to participate in the pathogenesis of MS and EAE by virtue of their ability to help in the formation of ectopic lymphoid follicles in the inflamed CNS (46). Tr1 cells which are differentiated *in vitro* by culturing in the presence of TGF-β plus IL-27 show the Foxp3^−^IL-10^+^IFN-γ^+^ phenotype. Tr1 cells are known to play a significant role in the development of transplantation tolerance (47), but their exact role in EAE is not known. However, IL-27Rα^−/−^ mice are hypersusceptible to the development of EAE, possibly because of a lack of IL-27-mediated control of Th17, as well as the absence of Tr1-mediated suppression (48). There are several Th1- and Th17-associated molecules, which play an important role in the pathogenesis of EAE, and their deficiency affects the severity of the disease (Table 1).

**Table 1:** Susceptibility and severity of experimental autoimmune encephalomyelitis (EAE) in various mouse strains.

Mouse strains	EAE type	Pathology of the disease	Disease susceptibility	Reference
IL-12p35^−/−^	Classical	Mononuclear cells in the spinal cord	Susceptible	(27, 49)
IL-12p40^−/−^			Resistant	(27, 49)
IL-12Rβ2^−/−^	Classical	Mononuclear cells in the spinal cord	Severe EAE	(50)
STAT1^−/−^	Atypical	Macrophage and neutrophils in the brain and spinal cord	Hypersusceptible	(18, 19)
STAT1^−/−^.T-bet^−/−^	Atypical	Macrophage and neutrophils in the brain and spinal cord	Less severe EAE	(15, 18)
T-bet^−/−^			Resistant	(13, 18)
STAT4^−/−^	Classical	Reduced infiltration in the spinal cord	Resistant	(35, 51)
STAT6^−/−^	Classical	Mononuclear cells in the spinal cord	Severe EAE	(51)
TNF-α^−/−^	Classical	Mononuclear cells in the spinal cord	Delayed but comparable severity	(52)
TNFR-1^−/−^	Classical	Mononuclear cells in the spinal cord	Less severe EAE	(53)
TNFR-2^−/−^	Classical	Mononuclear cells in the spinal cord	Severe EAE	(53)
TNFR1/2^−/−^	Classical	Mononuclear cells in the spinal cord	Severe EAE	(53)
IFN-γ^−/−^	Atypical	Predominantly neutrophils in the brain-stem and cerebellum	Hypersusceptible	(19)
IFN-γR^−/−^	Atypical	Predominantly neutrophils in the brain-stem and cerebellum	Hypersusceptible	(54)
IL-23p19^−/−^			Completely resistant	(23, 25)
IL-23R^−/−^			Completely resistant	(55)
IL-6^−/−^			Completely resistant	(56)
GM-CSF^−/−^			Completely resistant	(57)
IL-17A^−/−^	Classical	Mononuclear cells in the spinal cord	Delayed but comparable severity	(58)
IL-17F^−/−^	Classical	Mononuclear cells in the spinal cord	Susceptible	(58)
IL-21^−/−^	Classical	Mononuclear cells in the spinal cord	Susceptible	(59, 60)
IL-21R^−/−^	Classical	Mononuclear cells in the spinal cord	Susceptible	(59, 60)
IL-22^−/−^	Classical		Susceptible	(61)
IL-27Rα^−/−^	Classical		Hypersusceptible	(48)

## Migration of Myelin-Specific CD4^+^ T Cells into the CNS During Inflammation and Autoimmunity

Various cellular and molecular interactions help in maintaining the blood–brain barrier (BBB) integrity and immune quiescence into the CNS. It has been reported that astrocytes secrete Sonic hedgehog and endothelial cells (ECs) express Hedgehog receptors, and interaction of these receptor ligand promote the BBB formation and integrity (62). Migration of immune cells into the CNS parenchyma is a highly regulated process which occurs at various anatomical sites of the CNS, such as at the choroid plexus of the blood–CSF barrier, as well as across the BBB at post-capillary venules. The transmigration across the BBB is a very dynamic process and depends on a series of sequential and interdependent steps constituting tethering and rolling of immune cells, chemokine-induced activation, followed by polarization, crawling, the arrest of immune cells, and finally diapedesis of cells across the BBB ECs.

Intravital microscopic analysis of encephalitogenic T cell interactions with inflamed brain and spinal cord microvessels have revealed that the P-selectin glycoprotein ligand (PSGL-1)–P/E-selectin interaction mediates the initial rolling and tethering of CD4^+^ and CD8^+^ T cells (63). However, deficiencies of E- and P-selectin or PSGL-1 do not protect mice from EAE (64, 65), suggesting the redundant roles of these molecules during neuroinflammation. Followed by tethering, the α_4_β_1_-integrin on T cells interacts with endothelial vascular cell adhesion molecule 1 (VCAM-1) and is required for firm adhesion of T cells (66). Further studies are needed to clarify whether encephalitogenic T cells use alternative molecules for rolling and tethering onto the inflamed BBB.

The G protein-coupled receptors, such as chemokines and eicosanoids displayed on the luminal surface of the BBB ECs, trigger the integrin activation that leads to T cell firm arrest on the vascular endothelium. During EAE, ECs are shown to express CCL2, CCL19, and CCL21, which mediate firm arrest of CCR2^+^ monocytes and DCs, and CCR7^+^CD4^+^ T cells (67). However, the exact role of these interactions in the transendothelial migration (TEM) of encephalitogenic CD4^+^ T cells remains to be determined. Mice that overexpress CCL19, CCL21, or CXCL10 molecules in the CNS do not show hypersusceptibility to the EAE development (68, 69), suggesting that the functions of these chemokine interactions are tightly regulated. Chemokine receptor-induced signaling leads to a conformational change in the integrin molecules on CD4^+^ T cells, and which causes an increase in their affinity for their cognate ligands. Inflamed endothelial vessels in the CNS parenchyma upregulate the expression of the intercellular adhesion molecule 1 (ICAM-1) and VCAM-1, and their respective ligands, α_L_β_2_ [lymphocyte function-associated antigen 1 (LFA-1)], and α_4_β_1_ [very late antigen 4 (VLA-4)] integrins, are expressed on the encephalitogenic CD4^+^ T cells (70). Multiple investigators have shown that LFA-1–ICAM-1 and VLA-4–VCAM-1 interactions are critically involved in the firm arrest of CD4^+^ T cells onto the inflamed cerebral vessels or primary brain EC monolayers (71). Moreover, LFA-1–ICAM-1 interactions dictate the polarization and crawling of CD4^+^ T cells onto the inflamed vessels ECs, but VLA-4–VCAM-1 interactions do not (66). The anti-α4-integrin antibody, natalizumab has been approved for the treatment of relapsing-remitting MS (72). Interestingly, α_4_β_1_-integrin–VCAM-1 interaction arrest encephalitogenic Th1 cells onto the spinal cord microvessels, whereas LFA-1–ICAM-1/2 regulates Th17 adhesion to the endothelial barrier in the brain. This suggests that Th1 cells preferentially use α4-integrin, whereas Th17 transmigration across the BBB is α4-integrin independent (73, 74). Other cell adhesion molecules such as activated leukocyte cell adhesion molecule and melanoma cell adhesion molecule (MCAM) are also known to regulate transmigration of the CD4^+^ and CD8^+^ T cells and CNS autoimmunity (75–77). The MCAM expression in lymphocytes are associated with GM-CSF, IL-22, and IL-17A/IFN-γ coproducing Th17 cells (75), and antibody-mediated blocking of MCAM controls the CNS autoimmunity (75, 77). Recently, α_v_β_3_-integrin has been shown to control Th17 migration into the CNS, and a lack of β_3_ subunits ameliorates EAE (78). A genetic deficiency or functional blocking of most of the integrins and their ligands have yielded varied results in controlling the disease. These discrepancies might be due to a difference in the induction of EAE models, wherein the contribution of Th1 and Th17 varied to a significant extent. Live cell imaging studies of TEM across ICAM-1 and ICAM-2-deficient brain endothelial monolayers have revealed the presence of an alternative pathway for CD4^+^ T cell diapedesis (79).

Once arrested, CD4^+^ T cells start crawling on inflamed CNS microvessels to search the sites for diapedesis. TEM of CD4^+^ T cells across the BBB can occur through both, intercellular junctions and the cell body, known as paracellular and transcellular migration, respectively (80, 81). However, the physiological factors that dictate the choice of the transmigration route are not known, and thus forming an active area of investigation. Several studies have demonstrated that during TEM there is a fast and very dynamic remodeling of the endothelial junctional proteins that guide the migration of CD4^+^ T cells through paracellular route (82). The involvement of some of the adhesion molecules and junctional proteins such as PECAM-1, CD99, Claudin-5, VE-cadherin, and JAMs in the regulation of paracellular TEM has been very well studied (83). Upon attachment of the CD4^+^ T cells to the apical surface of inflamed brain ECs, a rapid clustering of ICAM-1 and VCAM-1 occurs around the transmigrating CD4^+^ T cells, resulting in the formation of transmigratory cups enriched with actin filaments (84). These clustering events trigger the various signaling pathways, leading to generation of an intracellular calcium flux, phosphorylation of key molecules that regulate the actin cytoskeleton, and production of reactive oxygen and nitric oxide species, which ultimately result in junctional disassembly (85). Numerous factors such as shear force, cytokine-induced inflammatory changes in the brain ECs, type of lymphocytes, and levels of junctional tightness have been hypothesized as the potential factors that regulate transcellular TEM of cells at the BBB. A recent study showed that cytokine-induced increased levels of ICAM-1 on the apical surface of primary mouse brain microvascular cell monolayers promote the transcellular TEM of CD4^+^ T cells possibly because of high occupancy of its receptor LFA-1 on CD4^+^ T cells (80, 81). In addition, overexpression of the C-terminal deletion mutant form of ICAM-1 in primary brain endothelial monolayers inhibits the TEM of leukocytes by reducing transcellular migration (86). Recently, the critical role of the lateral border recycling compartment, a recently identified endothelial specific subcellular compartment (enriched with PECAM-1 and CD99), have been shown to support both paracellular and transcellular TEM (87). The caveolin-rich transmigratory cups that surround the migrating CD4^+^ T cells have also been associated with transcellular TEM (88). While transcellular migration is impaired in caveolin-1-deficient ECs, they show higher paracellular TEM, suggesting that in the absence of one pathway another route can compensate (88). Similarly, whether a lack of paracellular migration at the endothelial monolayers of high barrier tightness, such as the BBB favors the transcellular route remains to be determined.

After crossing the endothelial vessels of the BBB, CD4^+^ T cells encounter the glial (glia limitans) basement membrane, and breaching this acellular structure represents the final step of trafficking into the CNS. The endothelial basement membrane at the BBB is characterized by the presence of laminin α_4_ and α_5_. It has been demonstrated that encephalitogenic CD4^+^ T cells cross the endothelial basement membrane through α_6_β_1_-integrin–laminin α_4_ interactions (89). On the other hand, laminin α_5_ in the endothelial basement membrane inhibits migration (89). Under physiological conditions, CXCL12 is abundantly expressed on the abluminal surface of brain endothelial microvessels, which inhibits the migration of CXCR4^+^ leukocytes into the CNS parenchyma (90). The cytokine-induced CXCR7 expression on these endothelial microvessels changes the localization of CXCL12 to the luminal surface, resulting in TEM of CXCR4^+^ leukocytes at the peak of the EAE (91). In contrast to the endothelial basement membrane, the glia limitans is enriched with laminin α1 and α2. Since encephalitogenic CD4^+^ T cells do not interact with laminin α1 and α2, they depend on matrix metalloproteinases (MMPs) to cross the basement membranes. Various types of MMPs, such as MMP-2, MMP-7, MMP-8, and MMP-9, have been identified in the CSF and lesions of MS and EAE. During EAE, MMP2 and MMP9 expression are specifically increased, and their combined action is positively correlated with the migration of CD4^+^ T cells across glia limitans (92). One of the targets of MMP2 and MMP9 is β-dystroglycan, a receptor which anchors astrocytic endfeet to the parenchymal basement membrane, leading to secretion of chemokines by the astrocytes at the glia limitans (93). Meningeal inflammation actively controls local CD4^+^ T cell reactivation and transmigration into the CNS. It has been recently shown that Th17-derived IL-17 and IL-22 activate meningeal stromal cells, which support the *de novo* IL-17 responses in the meninges (94). Interestingly, a finding has extended our current view about the role of T-bet beyond the generation of pathogenic Th1/Th17 cells and showed that T-bet expressing NKp46^+^ innate lymphoid cells (ILCs) promote meningeal inflammation and regulate EAE development by supporting Th17 migration into the CNS (95). Thus, the relay of coordinated signaling induced by cytokines, chemokines, and cell adhesion molecules in the ECs of the BBB and migrating CD4^+^ T cells orchestrate the multistep migration of encephalitogenic CD4^+^ T cells into the CNS parenchyma.

## Future Perspective

Both genetics and environmental factors cooperate to program auto-reactive CD4^+^ T cells to perform both pathogenic and regulatory functions during the course of autoimmune CNS pathologies. Recent evidence has suggested that the phenotype and functions of pathogenic Th1, Th17, and regulatory Tregs cells are regulated at various anatomic and physiological levels. The APCs in the periphery, tertiary lymphoid structures, stromal cells, and subsets of ILCs in the meninges, ECs at the BBB, and various CNS resident and infiltrating cells in the CNS parenchyma tightly control the activation, differentiation, and migration of CD4^+^ T cells that dictate the induction, maintenance, and resolution of autoimmune neuroinflammation. While considerable evidence already links Th1 and Th17 cells to the pathology of CNS autoimmunity, this list of cells continues to grow with recently identified subsets of CD4^+^ T cells, the IL-9-secreting Th9 cells, and IL-10-secreting Foxp3^−^ Tr1 cells (44, 96). However, the exact role of these cells and their associated molecules on the BBB, CNS resident cells, and other effector and regulatory leukocytes in the inflamed CNS parenchyma, and their overall impact in shaping neuroinflammation warrants further investigation. The ECs of the BBB have been recently shown to promote antigen-specific Th1 and Th17 migration through myelin-antigen presentation (97). However, the qualitative and quantitative differences in the regulation of transmigration of Th1, Th17, Th9, Tregs, and Tr1 cells across the BBB is not known and needs further attention. Experiments with knockout mice have revealed a great deal of information about the role of CD4^+^ T cell subsets and their lineage-associated transcription factors, cytokines, and homing receptors in the induction and propagation of CNS inflammation. The complete resistance of EAE in mice that lack T-bet, RORγt, IL-23R, and GM-CSF is attributed to the absence of pathogenic Th1 and Th17 functions (21, 26). However, these molecules are not exclusively expressed in CD4^+^ T cells, and the contribution of other myeloid and lymphoid cells, including subsets of γδ T cells, ILC1 and ILC3 that express/respond to these molecules, needs to be further investigated. Therefore, to better understand the pathophysiology of CD4^+^ T cells in autoimmune CNS diseases, we need to dissect out the contributions made by the other cell types that share the transcription factors, cytokines, and homing receptors of CD4^+^ T cell lineages. A reductionist approach may help in probing the exact role of CD4^+^ T cell subsets through the course of CNS inflammation and autoimmunity.

## Author Contributions

SS and GL planned and wrote the manuscript.

## Conflict of Interest Statement

The authors declare that the research was conducted in the absence of any commercial or financial relationships that could be construed as a potential conflict of interest.

## Abbreviations

APCs, antigen-presenting cells; BBB, blood–brain barrier; CFA, complete Freund’s adjuvant; CSF, cerebrospinal fluid; CNS, central nervous system; EAE, experimental autoimmune encephalomyelitis; ECs, endothelial cells; ICAM-1, intercellular adhesion molecule 1; ILCs, innate lymphoid cells; LFA-1, lymphocyte function-associated antigen 1; MOG_35–55_, myelin oligodendrocyte glycoprotein peptide amino acid 35–55; MS, multiple sclerosis; TEM, transendothelial migration; Tregs, Foxp3+ regulatory T cells; Tr1 cells, T regulatory 1 cells; VCAM-1, vascular cell adhesion molecule 1; VLA-4, very late antigen 4.
